# A longitudinal study of the effect of temperature modification in full-scale anaerobic digesters – dataset combining 16S rDNA gene sequencing, metagenomics, and metabolomics data

**DOI:** 10.1016/j.dib.2022.107960

**Published:** 2022-02-18

**Authors:** Francesc Puig-Castellví, Cédric Midoux, Angéline Guenne, Delphine Conteau, Oscar Franchi, Chrystelle Bureau, Céline Madigou, Delphine Jouan-Rimbaud Bouveresse, Pablo Kroff, Laurent Mazéas, Douglas N. Rutledge, Gilberte Gaval, Olivier Chapleur

**Affiliations:** a Université Paris-Saclay, INRAE, AgroParisTech, UMR SayFood, Paris 75005, France; b Université Paris-Saclay, INRAE, PRocédés Biotechnologiques au Service de l'Environnement, Antony 92160, France; c Université Paris-Saclay, INRAE, MaIAGE, Jouy-en-Josas 78350, France; d Université Paris-Saclay, INRAE, BioinfOmics, MIGALE Bioinformatics Facility, Jouy-en-Josas 78350 France; e Suez-CIRSEE, Le Pecq 78230, France; f Universidad Adolfo Ibanez, Facultad de ingeniería y ciencias, Viña del mar 2520000, Chile; g Université Paris-Saclay, AgroParisTech, INRAE, UMR PNCA, 75005 Paris, France; h National Wine and Grape Industry Centre, Charles Sturt University, Wagga 2650, Australia

**Keywords:** Anaerobic digestion, 16S sequencing, Multiomic, Metagenomics, Metabolomics, Methanization, Temperature, energy production

## Abstract

Data in this article provides detailed information on the microbial dynamics and degradation performances in two full-scale anaerobic digesters operated in parallel for 476 days. One of them was kept at 35 °C for the whole experiment, while the other was submitted to sub-mesophilic (25 °C) conditions between days 123 and 373. Sludge samples were collected from both digesters at days 0, 80, 177, 218, 281, 353, and 462. The provided data include the operational conditions of the digesters and the characterization of the sludge samples at the physicochemical level, indicative of the digesters’ degradation performance. It also includes the characterization of the sludge samples at the multiomics level (16S rRNA gene sequencing, metagenomics, and metabolomics profiling), to decipher the changes in the microbial structure and molecular activity. The 16S rDNA gene sequencing, metagenomics, and metabolomics data were generated using an IonTorrent PGM sequencer, an Illumina NextSeq 500 sequencer, and LTQ-Orbitrap XL mass spectrometer respectively. The 16S rDNA gene raw data and the metagenomics data have been deposited in the BioProject PRJEB49115, in the ENA database (https://www.ebi.ac.uk/ena/browser/view/PRJEB49115). The metabolomics data has been deposited at the Metabolomics Workbench, with study id ST002004 (DOI: 10.21228/M8JM6B). The data can be used as a source for comparisons with other studies working with data from full-scale anaerobic digesters, especially for those investigating the effect of the temperature modification. The data is associated with the research article “*Metataxonomics, metagenomics, and metabolomics analysis of the influence of temperature modification in full-scale anaerobic digesters*” (Puig-Castellví et al [1]).

## Specifications Table

**Table:** 

Subject area	Biology
More specific subject area	Microbial ecology of anaerobic digestion
Type of data	Table, raw sequencing data, metabolomics data
How data was acquired	The biogas flow was monitored online using a Proline t-mass sensor (Reinach, Endress+Hauser, Switzerland). The percentage of methane was measured using the Geotech GA2000 portable gas analyzer (Geotech GA2000, Coventry, UK). Total ammonium (N—NH_4_^+^) concentration and Chemical Oxygen Demand (COD) were determined using Hach kits. Total alkalinity and volatile fatty acids (TAC and VFA, respectively) were determined with the Hach automatic tritator model EZ7250. Total and volatile solids (TS and VS, respectively) were determined according to the Standard Methods for the Examination of Water and Wastewater (APHA, 1995). All parameters were measured twice a week except for total ammonium, which measurements were discontinued after day 336. Weekly average is provided. 16S rRNA gene sequencing was carried out with the Ion Torrent Personal Genome Machine. Metagenomics sequencing was performed with the Illumina NextSeq 500. Metabolomics data was acquired with the LTQ-Orbitrap XL mass spectrometer.
Data format	Raw, analyzed
Parameters for data collection	The full-scale experiment was carried out in France in a wastewater treatment plant (WWTP) with a population equivalent of 190,000. The two 3000 m^3^ full-scale anaerobic digesters (A and B) were operated in parallel. They were fed with a mixed substrate composed of sewage sludges from wastewater treatment plants and waste from slaughterhouses. Sludge was collected from two full-scale digesters at days 0, 80, 177, 218, 281, 353, and 462.
Description of data collection	Sludge was collected in 50-mL sterile Falcon tubes and immediately transported on dry ice to INRAE, where they were frozen at −80 °C.
Data source location	Antony, France
Data accessibility	Operational and performance data are available in the article. The 16S rDNA gene sequencing data and the metagenomics data have been deposited in the bioproject PRJEB49115 (https://www.ebi.ac.uk/ena/browser/view/PRJEB49115), with the taxonomy ID 1,263,854 (anaerobic digester metagenome). The metabolomics data have been deposited in the Metabolomics Workbench, with study id ST002004 (DOI: 10.21228/M8JM6B).
Related research article	Metataxonomics, metagenomics, and metabolomics analysis of the influence of temperature modification in full-scale anaerobic digesters. Puig-Castellví, F., Midoux, C., Guenne, A., Conteau, D., Franchi, O., Bureau, C., Madigou, C., Jouan-Rimbaud Bouveresse, D., Kroff, P., Mazéas, L., Rutledge, D.N., Gaval, G., Chapleur. O. Bioresource Technology.

## Value of the Data

• These data provide a link between the operational temperature in anaerobic digesters and their performance (biogas production, total ammonia, pH, volatile fatty acids, total alkalinity, total solids, volatile solids, and chemical oxygen demand), their microbial structure, the molecular functions of their microbial communities, and their metabolic profiles.
• All data presented in this paper can be used as a source for comparisons with other studies working with data from full-scale anaerobic digesters. It is specifically interesting for those investigating the effect of the temperature modification. It can also be used for seeking more associations between the operational, the physicochemical and the ‘omics data.
• Metagenomics and 16S data can be used to identify microorganisms and molecular functions characteristic of the different working temperatures. Metabolomics data reveals the metabolic changes in the digesters resulting from the interaction of the microbial community with the digesters’ substrate. Accessibility to the multiomics dataset and detailed associated metadata will allow researchers to perform new analyzes They can also use this data for the development of new methods to integrate and visualize multiomics data.
• We provide access to omics data from full-scale digesters, which is very limited. In particular metabolomics has been an underexplored technique in the area of anaerobic digestion despite its large potential for characterization of biological material.
• The operational setup of having two full-scale digesters working in parallel can minimize the effect of external factors (i.e. variability of the substrate over time, weather). It is useful to extract the pure contribution of the studied factor. Accessibility to data obtained from this type of operational setups is not common either.

## 1 Data Description

Anaerobic digestion is normally operated at mesophilic (30–40 °C) or thermophilic (50–60 °C) conditions (Jain et al., [2]). Anaerobic digestions at higher temperatures are faster and produce higher biogas yields. They present an accelerated microbial metabolism in general, including hydrolysis and acidogenesis of recalcitrant feedstock compared to mesophilic conditions (Pasalari et al.*,* [3]). Nevertheless, sub-mesophilic conditions are not rare for full-scale digestion, as they can derive from system malfunction but also from the desire to limit heating cost. In this work, we investigated the physicochemical and omics changes derived from the modification of the temperature in full-scale anaerobic digesters.

Table S1 presents the operational data and physicochemical data measured throughout all the experiment (temperature, methane production, pH, N—NH_4_^+^, VFA, TAC, TS, VS, COD), averaged by week.

The sequencing data was deposited as fastq.gz files in the European Nucleotide Archive. This data includes metagenomics shotgun data as paired files generated with an Illumina Nextseq 500, and 16S rRNA gene sequencing with Ion Torrent PGM. Deposited metagenomics files comprise data from the two reactors and 5 time points (days 80, 177, 218, 281 and 462), while the 16S rRNA gene sequencing data covered all the time points.

The metabolomics data were deposited as mzXML files in the Metabolomics workbench. These data consist of 56 biological samples (4 replicates for each time and reactor), 11 quality controls samples and 11 blanks.

## 2 Experimental Design, Materials and Methods

### 2.1 Experimental design and sampling

The full-scale experiment was carried out in France in a wastewater treatment plant (WWTP) with a population equivalent of 190,000. The two 3000 m^3^ full-scale anaerobic digesters (A and B) were operated in parallel. They were fed with a mixed substrate composed of sewage sludges from wastewater treatment plants and wastes from slaughterhouses. During the time of the experiment, both reactors worked at half capacity due to a constraint in the availability of substrate to treat.

The experimental period lasted 462 days. For the first four months of study, the two reactors operated at 35 °C. Reactor A was kept at this temperature during all the experiment. For digester B, the temperature was decreased abruptly to 30 °C on the 123th day and maintained at this level for 2 hydraulic retention times (HRTs) until the 190th day. Then, the temperature was modified to 25 °C and maintained for 3 HRTs (between the 191st and the 373th days). Finally, the heating of reactor B was increased from day 374 onwards to reach the initial temperature of 35 °C.

Sludge samples from the two reactors were collected at days 0, 80, 177, 218, 281, 353, and 462. Sludge was collected in 50-mL sterile Falcon tubes and immediately transported on dry ice to INRAE, where they were frozen at −80 °C.

### 2.2 DNA extraction

For every sample, the total DNA was extracted from 0.2 g of sludge using the DNeasy PowerSoil DNA Elution Kit (Qiagen, Hilden, Germany) following the manufacturer's instruction. DNA was quantified with the Qubit 2.0 Fluorometer (dsDNA BR Assay Kit, Invitrogen, USA).

### 2.3 16S amplification and sequencing

The DNA extracted was used for the amplification of the bacterial and archaeal hypervariable region V4–V5 of the 16S rRNA gene with the primers 515F (5′-GTGYCAGCMGCCGCGGTA-3′). and 928R (5′-CCCCGYCAATTCMTTTRAGT-3′) as described by Puig-Castellví et al [4]. Sequencing was performed on the Ion Torrent Personal Genome Machine using Ion 316 chip and the Ion PGM Sequencing 400 Kit.

### 2.4 Metagenomics read processing

The shotgun sequencing of the genomic DNA of samples collected on days 80, 177, 218, 281 and 462 was carried out.

Libraries were prepared using 200 ng DNA with the TruSeq NGS Library Prep Kit from Westburg (WB9024) following the manufacturer's instructions. Libraries were amplified using the Kapa HiFi DNA polymerase (10 cycles). Library quality was checked using the Bioanalyzer system from Agilent and sequencing was done using the Illumina NextSeq 500, generating 150 bp paired end reads (NextSeq 500 High Output Kit 300 cycles). Demultiplexing was done (bcl2fastq2–2.18.12) and adapters removed (Cutadapt1.15), only reads longer than 10 pb were kept for analysis. The quality of the data was checked with FastQC v0.11.5.

### 2.5 Metabolite extraction

For every sample, four aliquots with 0.75 mL of sludge were prepared in 2 mL Eppendorf tubes. Then, 1 mL of phosphate buffer saline was added into each tube and the sludge volumes were washed by centrifuging them at 15,500 rcf for 3 min at 4 °C. This process was repeated once. The samples were freeze-dried overnight and, for every sample, 20 mg of dried sludge were transferred in new Eppendorf tubes. Then, the dried sludge powders were resuspended in 1 mL of a cold ddH_2_O: MeOH mixture (1:1) containing 2.5 mM PIPES. Samples were sonicated for 6 min, and submerged in liquid nitrogen. After one minute, the samples were thawed in ice. The cold-shock step was repeated twice. Afterwards, the samples were agitated for 15 min at 420 rpm in an orbital shaker. Following this, the samples were centrifuged at 15,500 rcf for 3 min at 4 °C and the supernatants were collected. The supernatants were immediately filtered with 0.45 µM Nylon filters and freeze-dried overnight. The dried extracts were left at −80 °C until the high performance liquid chromatography coupled to mass spectrometry (HPLC-MS) analyzes.

### 2.6 HPLC-MS analysis

The dried extracts were resuspended in 1 mL of a H_2_O: ACN mixture (1:1). Quality Control samples (QCs) composed of a pool of all samples were prepared. The instrumentation consisted of an Accela 1250 pump system connected to a LTQ-Orbitrap XL mass spectrometer (Thermo Scientific, Waltham, MA, USA) operated in positive electrospray ionization mode (ESI+). The detection was performed in full scan over an m/z range from 50 to 800 at a resolution of 60,000. A NUCLEODUR HILIC column (100 mm × 2.1 mm ID, particle size 1.8 µm) provided with a NUCLEODUR HILIC guard column (4 mm × 2.1 mm ID, particle size 1.8 µm) (Macherey-Nagel, Düren, Germany) was used. The mobile phases were milli-Q acetonitrile (phase A) and water containing 50 mM ammonium acetate at pH 5.0 (phase B). For each sample, 10 µL were injected. The flow rate was set at 0.4 mL min^−1^. The HPLC gradient started at 5% B and held for 3 min, then increased at 40% B during 11 min. From minutes 14 to 24, B was linearly decreased to 5%. Finally, initial conditions were re-equilibrated in 5 min, resulting in a total run time of 29 min. To remove possible batch effects, samples were injected in random order. Moreover, Quality Controls (QCs) and blanks were injected every 8 samples. In total, 78 samples were injected (56 samples (2 reactors × 7 time-points × 4 replicates), 11 QCs and 11 blanks). The generated RAW HPLC-MS data were converted into mzXML-format files using MSConvert (ProteoWizard 3.0).

## Ethics Statement

The authors did not use animal or human experimental materials and thus are not subject to ethical concerns.

## CRediT authorship contribution statement

**Francesc Puig-Castellví:** Investigation, Data curation, Writing – original draft, Visualization. **Cédric Midoux:** Formal analysis, Data curation, Writing – original draft. **Angéline Guenne:** Investigation. **Delphine Conteau:** Conceptualization, Resources, Supervision. **Oscar Franchi:** Investigation. **Chrystelle Bureau:** Investigation. **Céline Madigou:** Investigation. **Delphine Jouan-Rimbaud Bouveresse:** Supervision. **Pablo Kroff:** Resources, Supervision, Funding acquisition. **Laurent Mazéas:** Methodology, Writing – review & editing, Supervision, Project administration, Funding acquisition. **Douglas N. Rutledge:** Methodology, Software, Supervision, Funding acquisition. **Gilberte Gaval:** Conceptualization, Supervision. **Olivier Chapleur:** Conceptualization, Methodology, Writing – review & editing, Supervision, Funding acquisition.

## Declaration of Competing Interest

The authors declare that they have no known competing financial interests or personal relationships which have or could be perceived to have influenced the work reported in this article.

## Appendix Supplementary materials

**Table 1:** Weekly average of the operational and physicochemical data (temperature, methane production, pH, N—NH_4_^+^, VFA, TAC, TS, VS, COD). Image, application 1
